# From theory to practice: Careful construction and validation of a predictive model for colostomy for colorectal cancer peristomal irritant dermatitis

**DOI:** 10.1097/MD.0000000000046266

**Published:** 2025-11-28

**Authors:** Fei An, Lin Gui, Yuting Li, Minjing Cheng

**Affiliations:** a Department of Gastrointestinal Surgery, The Third Hospital of Hebei Medical University, Shijiazhuang City, Hebei Province, China.

**Keywords:** anxiety, colorectal cancer, enterostomy, peristomal irritant dermatitis, predictive model

## Abstract

Peristomal irritant dermatitis (PIAD) is a common and difficult complication after stoma surgery, which brings both physical and psychological torture and seriously interferes with the normal recovery process and daily life. In-depth analysis of the influencing factors of PIAD and the establishment of a scientific and efficient prediction model have become the most urgent task to improve the quality of stoma care and the outcome of patients. Exploration of risk factors for postoperative development of PIAD in patients with colostomy for colorectal cancer and development of relevant predictive models. A total of 272 patients were collected in the study, of whom 58 had stoma prolapse; the patients enrolled in this study were randomly divided into a training set and a validation set according to a ratio of 7:3, of whom 190 were in the training set and 82 in the validation set. The patients’ past medical history, stoma-related information and information related to the hospitalization period were collected separately to study the correlation factors affecting the emergence of PIAD in patients and to establish a prediction model. The potentially relevant factors were included in a 1-way logistic regression, and after analyzing the results: regular stoma clinic review, stoma site, ostomy flange fit, hypoproteinemia, anxiety, and radiotherapy were potential risk factors for PIAD, *P* < .05; The data obtained were further included in a multifactorial review: regular stoma clinic review, stoma site, ostomy flange fit or not, hypoproteinaemia, and anxiety were independent risk factors for PIAD (*P* < .05). This model provides a powerful tool for clinicians to identify patients at high risk of postoperative PIAD, which can help to take targeted preventive and interventional measures before the occurrence of dermatitis.

## 1. Introduction

Colorectal cancer, as a highly prevalent malignant tumor worldwide, is a serious threat to human health. Surgical resection combined with stoma is one of the most important means of treatment for colorectal cancer, which can effectively prolong the survival period of patients and bring hope of survival to many patients.^[1–4]^ With the wide application of stoma technology at home and abroad, the number of patients undergoing stoma surgery for colorectal cancer continues to climb, and their postoperative quality of life has become an increasing clinical concern.^[5,6]^ Overseas, developed countries such as the United States and Europe have taken the lead in conducting a large number of studies on stoma care. For example, a multicentre study in the United States followed thousands of patients with stoma over a long period of time and found that the annual incidence of peristomal irritant dermatitis (PIAD) in patients with stoma for colorectal cancer was as high as 26% to 50%, which is a statistic that highlights the seriousness of the problem of PIAD.^[7,8]^ Foreign scholars have actively explored the pathogenesis of PIAD, and through advanced skin physiological testing technology, they have clarified the correlation between the composition of excreta, the wearing time of stoma appliances and other factors and the occurrence of PIAD. Predictive modeling in healthcare is essential not only for preventing patient complications, such as PIAD, but also for anticipating hospital resource demands. By identifying high-risk patients early, hospitals can better plan staffing, equipment, and bed capacity. This develops a strong link between individual risk prediction and system-level demand forecasting, ultimately improving both patient outcomes and healthcare efficiency.^[9]^ Meanwhile, some teams have tried to construct prediction models, such as using logistic regression analysis to screen out multiple risk factors, but the general applicability and prediction accuracy of the models still need to be improved.^[10,11]^ Domestic research in the field of colostomy for colorectal cancer stoma care has been equally successful. In recent years, many medical centers have actively carried out clinical research to deeply analyze the characteristics of domestic patients. Studies have shown that due to the influence of dietary habits, living environment and other factors, the incidence pattern of PIAD in domestic patients differs from that of foreign countries to a certain extent.^[12]^ On the basis of traditional nursing experience, domestic scholars have introduced information management tools and tried to construct prediction models with the help of big data analysis. However, most of these models are still in the preliminary stage of exploration, and have not yet formed a set of systematic, accurate and mature solutions that can be widely used in clinical practice.

The current stoma care programmes have obvious shortcomings in PIAD risk assessment. Most programmes rely on traditional experience and lack objective and quantitative assessment indicators, making it difficult for caregivers to accurately identify high-risk patients with PIAD at an early stage, and the pertinence and effectiveness of preventive measures are greatly reduced. Despite the continuous innovation of stoma technology and nursing concepts, the pathogenesis of PIAD has not been fully clarified, and the construction of prediction models lags far behind the actual clinical needs. In this context, the careful construction and validation of a set of scientific and efficient PIAD prediction model for colostomy for colorectal cancer is of pivotal significance to enhance the quality of care and improve the prognosis of patients with colorectal cancer stoma in China and even in the world, and it is expected to fill the key gaps in the field both at home and abroad, and to promote the stoma care from the traditional empirical model to the direction of precision and intelligence.

## 2. Materials and methods

### 2.1. Research object

The subjects of this study were patients who consulted the Department of Gastrointestinal Surgery of the Third Hospital of Hebei Medical University from January 2021 to June 2024, were diagnosed with colorectal cancer and underwent stoma in our department. There were a total of 272 patients, including 161 males and 111 females. The patients were informed about the study and signed relevant informed consent forms with them. The study was approved by the Ethics Committee of our hospital. Number: W-2025-034-1.

### 2.2. Research methods

This study used a retrospective case–control study. A total of 272 patients were collected, of which 58 patients presented with PIAD. The patients’ past medical history, stoma-related information and information during hospitalization were collected separately to study the risk factors affecting the development of PIAD after colostomy for colorectal cancer.

### 2.3. Collection of indicators

Gender, age, BMI, history of smoking, history of alcohol consumption, hypertension, diabetes mellitus, coronary artery disease, radiotherapy, regular stoma clinic reviews, stoma position, ostomy flange fit anemia, anxiety and occurrence of hypoproteinaemia.

### 2.4. Inclusion and exclusion criteria

#### 2.4.1. Inclusion criteria

(1) Patients who underwent ostomy and whose postoperative pathological diagnosis met the criteria of the Chinese Colorectal Cancer Diagnostic and Treatment Code (2023 edition);
(2) Having an ostomy for a period of more than 1 month;
(3) Being ≥ 18 years old, being aware of the disease, and having the ability to communicate in normal language;
(4) Having voluntarily participated in the study and signing the informed consent form;
(5) Having complete clinical data.

#### 2.4.2. Exclusion criteria

(1) Patients with other malignant tumors;
(2) Those with other serious complications of the heart, brain, kidney, lungs, etc;
(3) Those with previous psychiatric disorders or severe psycho-cognitive dysfunction.
(4) Patients who are non-compliant or refuse to participate in the study.

### 2.5. Interpretation of some of the validation indicators mentioned in this study

(1) Stoma location: i.e., the bowel selected for stoma, including colostomy and ileostomy.
(2) Ostomy bag: The ostomy bag is a container used to store excreta such as feces after the patient has undergone ostomy surgery. The ostomy bag chassis is part of the ostomy bag, and it mainly serves to fix the ostomy bag and protect the skin.
(3) Assessment of patient anxiety: We used the Self-Rating Anxiety Scale to assess the occurrence of postoperative depression in patients.

### 2.6. Statistical methods

The data was processed and statistically analyzed in this study using SPSS 25.0. Quantitative information that conformed to normal distribution was expressed as mean ± standard deviation, and differences between groups were analyzed using the independent samples t-test. Comparisons between groups that do not obey a normal distribution are made using nonparametric tests. Data for qualitative information were expressed as number of cases and percentages, and the chi-square test was used to determine if there were differences between groups. The patients were first randomly divided into a training set and a validation set in a 7:3 ratio. Then, based on the occurrence of PIAD in the training set of patients and various clinically relevant indicators and other factors, an analysis was carried out. Potential risk factors for the development of PIAD were subsequently identified based on a 1-way logistic regression analysis of the collected data. For the univariate analysis, exposure factors with *P* ≤ .2 were selected and included in the multivariate analysis. Independent risk factors were derived with respect to the occurrence of PIAD, with *P* < .05 being considered a statistically significant difference. Subsequent internal validation of the model further confirmed the reliability of the predictive model derived in this study.

## 3. Results

### 3.1. Table of values assigned to the relevant indicators in this study

In this study, the variables were assigned values based on the categorization of variables such as gender, age, PIAD, Ostomy flange fit, and stoma site. For example, if the gender is female, the value 0 is assigned; if the gender is male, the value 1 is assigned. See Table 1 for details.

**Table 1:** Assignment table.

Name	Variable assignment and description
Gender	Female-0, Male-1
Age	<50 yr-0, ≥50 yr-1
PIAD	No-0, Yes-1
Ostomy flange fit	Fit well-0, Do not fit well-1
Stoma site	Colostomy-0, Ileostomy-1

PIAD = peristomal irritant dermatitis.

### 3.2. Table of baseline characteristics of patients in both groups

We randomly divided the patients included in this experimental study into a training set and a validation set in a ratio of 7:3. Among them, there were 190 in the training set and 82 in the validation set. The general information of the patients in the 2 sets was included in the statistical study, *P* > .05, and the differences in the baseline characteristics of the 2 sets were not statistically significant, as shown in Table 2.

**Table 2:** Comparison of baseline features between training and validation sets.

Variables	Total (n = 272)	test (n = 82)	train (n = 190)	Statistic	*P*
BMI	23.20 ± 2.84	23.42 ± 2.79	23.11 ± 2.86	*t* = 0.83	.407
PIAD, n (%)				χ² = 0.64	.423
0	214 (78.68)	67 (81.71)	147 (77.37)		
1	58 (21.32)	15 (18.29)	43 (22.63)		
Gender, n (%)				χ² = 3.02	.082
0	111 (40.81)	27 (32.93)	84 (44.21)		
1	161 (59.19)	55 (67.07)	106 (55.79)		
Age, n (%)				χ² = 1.22	.270
0	93 (34.19)	32 (39.02)	61 (32.11)		
1	179 (65.81)	50 (60.98)	129 (67.89)		
Smoking history, n (%)				χ² = 0.58	.446
0	204 (75.00)	64 (78.05)	140 (73.68)		
1	68 (25.00)	18 (21.95)	50 (26.32)		
Drinking history, n (%)				χ² = 3.01	.083
0	151 (55.51)	39 (47.56)	112 (58.95)		
1	121 (44.49)	43 (52.44)	78 (41.05)		
Hypertensive, n (%)				χ² = 0.17	.678
0	184 (67.65)	54 (65.85)	130 (68.42)		
1	88 (32.35)	28 (34.15)	60 (31.58)		
Diabetes mellitus, n (%)				χ² = 0.50	.479
0	222 (81.62)	69 (84.15)	153 (80.53)		
1	50 (18.38)	13 (15.85)	37 (19.47)		
Coronary heart disease, n (%)				χ² = 0.00	.983
0	239 (87.87)	72 (87.80)	167 (87.89)		
1	33 (12.13)	10 (12.20)	23 (12.11)		
Radiotherapy, n (%)				χ² = 0.01	.933
0	147 (54.04)	44 (53.66)	103 (54.21)		
1	125 (45.96)	38 (46.34)	87 (45.79)		
Regular stoma clinic reviews, n (%)				χ² = 0.12	.730
0	79 (29.04)	25 (30.49)	54 (28.42)		
1	193 (70.96)	57 (69.51)	136 (71.58)		
Stoma site, n (%)				χ² = 1.98	.160
0	112 (41.18)	39 (47.56)	73 (38.42)		
1	160 (58.82)	43 (52.44)	117 (61.58)		
Ostomy flange fit, n (%)				χ² = 0.09	.759
0	163 (59.93)	48 (58.54)	115 (60.53)		
1	109 (40.07)	34 (41.46)	75 (39.47)		
Anemic, n (%)				χ² = 0.02	.899
0	151 (55.51)	46 (56.10)	105 (55.26)		
1	121 (44.49)	36 (43.90)	85 (44.74)		
Hypoproteinemia n (%)				χ² = 0.37	.543
0	135 (49.63)	43 (52.44)	92 (48.42)		
1	137 (50.37)	39 (47.56)	98 (51.58)		
Anxiety, n (%)				χ² = 0.04	.835
0	175 (64.34)	52 (63.41)	123 (64.74)		
1	97 (35.66)	30 (36.59)	67 (35.26)		

BMI = body mass index, PIAD = peristomal irritant dermatitis.

### 3.3. One-way analysis of variance

The training set of 190 patients was included in the statistical analysis, of which 43 patients presented with PIAD.The potentially relevant factors were included in the univariate logistic regression, in which regular stoma clinic review, stoma site, ostomy flange fit or not, hypo-proteinaemia, anxiety, and radiotherapy were the potential risk factors for the development of PIAD in postoperative period in patients with colorectal cancer stoma, *P* < .05. See Table 3 for details.

**Table 3:** One-factor logistic regression analysis based on training set.

Variables	β	S.E	Z	*P*	OR (95% CI)
Gender					
0					1.00 (Reference)
1	0.12	0.35	0.35	.724	1.13 (0.57–2.25)
Age					
0					1.00 (Reference)
1	0.26	0.38	0.67	.503	1.29 (0.61–2.73)
Smoking history					
0					1.00 (Reference)
1	−0.21	0.41	−0.52	.605	0.81 (0.37–1.80)
Drinking history					
0					1.00 (Reference)
1	−0.08	0.35	−0.23	.818	0.92 (0.46–1.84)
Hypertensive					
0					1.00 (Reference)
1	0.06	0.37	0.16	.875	1.06 (0.51–2.19)
Diabetes mellitus					
0					1.00 (Reference)
1	0.47	0.41	1.14	.253	1.60 (0.71–3.58)
Coronary heart disease					
0					1.00 (Reference)
1	0.22	0.51	0.42	.673	1.24 (0.46–3.37)
Regular stoma clinic reviews					
0					1.00 (Reference)
1	−1.19	0.36	−3.28	.001	0.30 (0.15–0.62)
Stoma site					
0					1.00 (Reference)
1	0.91	0.40	2.28	.023	2.48 (1.14–5.40)
Ostomy flange fit					
0					1.00 (Reference)
1	1.25	0.36	3.45	<.001	3.48 (1.71–7.06)
Anemic					
0					1.00 (Reference)
1	−0.27	0.35	−0.78	.436	0.76 (0.38–1.52)
Hypoproteinemia					
0					1.00 (Reference)
1	1.43	0.40	3.59	.001	4.16 (1.91–9.07)
Anxiety					
0					1.00 (Reference)
1	1.12	0.36	3.13	.002	3.06 (1.52–6.15)
Radiotherapy					
0					1.00 (Reference)
1	1.16	0.37	3.15	.002	3.18 (1.55–6.52)
BMI	−0.04	0.06	−0.74	.461	0.96 (0.85–1.08)

BMI = body mass index, PIAD = peristomal irritant dermatitis.

### 3.4. Multifactorial analysis

The risk factors derived from the univariate analysis of this study were further included in the multivariate analysis, and the results showed that regular stoma clinic review, stoma site, ostomy flange fit or not, hypoproteinemia, and anxiety were the independent risk factors for postoperative development of PIAD in patients with colostomy for colorectal cancer, *P* < .05. See Table 4 for details.

**Table 4:** Multifactor logistic regression analysis based on training set.

Variables	β	S.E	Z	*P*	OR (95% CI)
Intercept	−4.53	0.90	−5.02	<.001	0.01 (0.00–0.06)
Regular stoma clinic reviews					
0					1.00 (Reference)
1	−1.15	0.46	−2.52	0.012	0.32 (0.13–0.77)
Stoma site					
0					1.00 (Reference)
1	1.06	0.49	2.15	0.032	2.88 (1.10–7.56)
Ostomy flange fit or not					
0					1.00 (Reference)
1	1.41	0.46	3.10	0.002	4.11 (1.68–10.05)
Hypoproteinemia					
0					1.00 (Reference)
1	2.13	0.52	4.07	<.001	8.44 (3.02–23.55)
Anxiety					
0					1.00 (Reference)
1	1.94	0.49	3.97	<.001	6.98 (2.67–18.21)

CI = confidence interval, OR = odds ratio, PIAD = peristomal irritant dermatitis.

### 3.5. Plotting of nomograms

**Figure 1.**
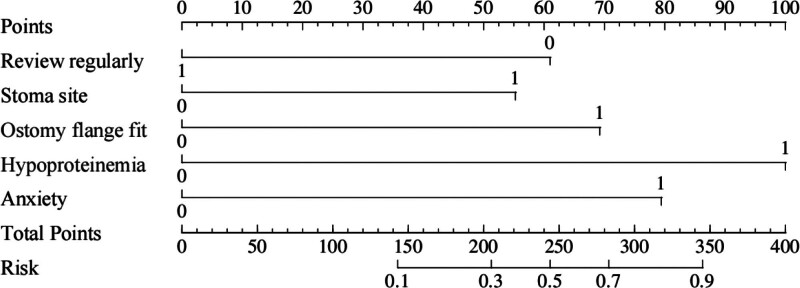
Nomogram prediction of risk of postoperative PIAD in patients with colostomy for colorectal cancer. PIAD = peristomal irritant dermatitis.

A nomogram of the risk of postoperative PIAD in patients with colostomy for colorectal cancer was constructed based on 5 independent predictors tested by multifactorial logistic regression analysis, as shown in Figure 1. A Nomo score was assigned to each independent risk factor, which was summed to give a total score based on the clinical characteristics of that patient, positioned on the Total points axis. A vertical line is made down the total score, and the value on the corresponding Risk axis is the probability of postoperative PIAD in that patient with a colostomy for colorectal cancer. The score for each independent predictor corresponds to the upper limit of the score for each independent predictor. The total score for each subject was the sum of each independent predictor score. The probability of developing PIAD was determined by the total score on the risk axis of developing PIAD after ostomy in colorectal cancer patients. The model was subsequently validated internally, and the internal validation was performed by repeated sampling of the nomogram 1000 times using the Bootstrap method in R software. The calibration curve is close to the ideal curve, indicating that the nomogram predicts the incidence of postoperative occurrence of PIAD in patients with colostomy for colorectal cancer with a high degree of agreement with the actual incidence, reflecting a good predictive performance, see

**Figure 2.**
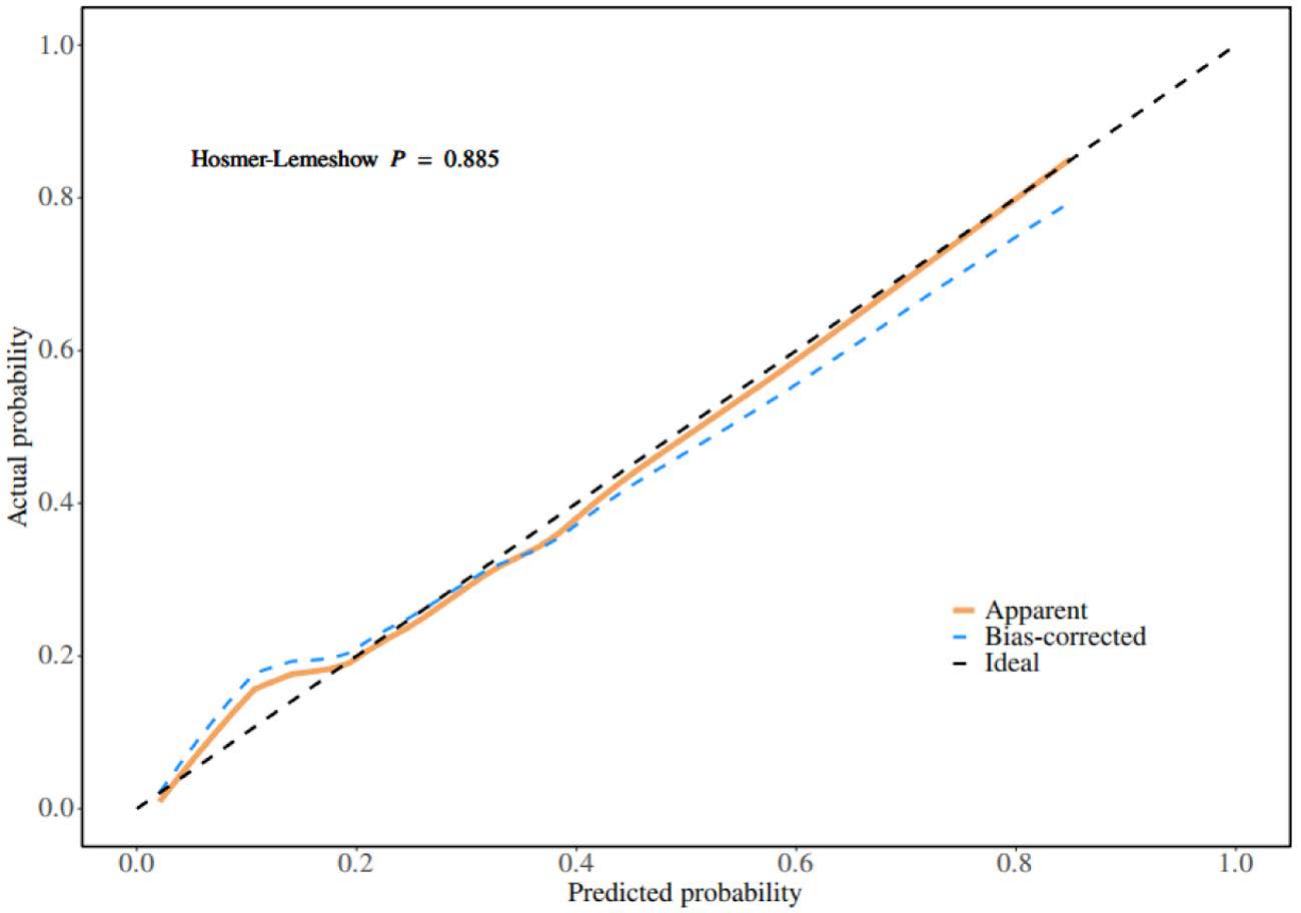
Internal validation of nomograms: calibration curves.

**Figure 3.**
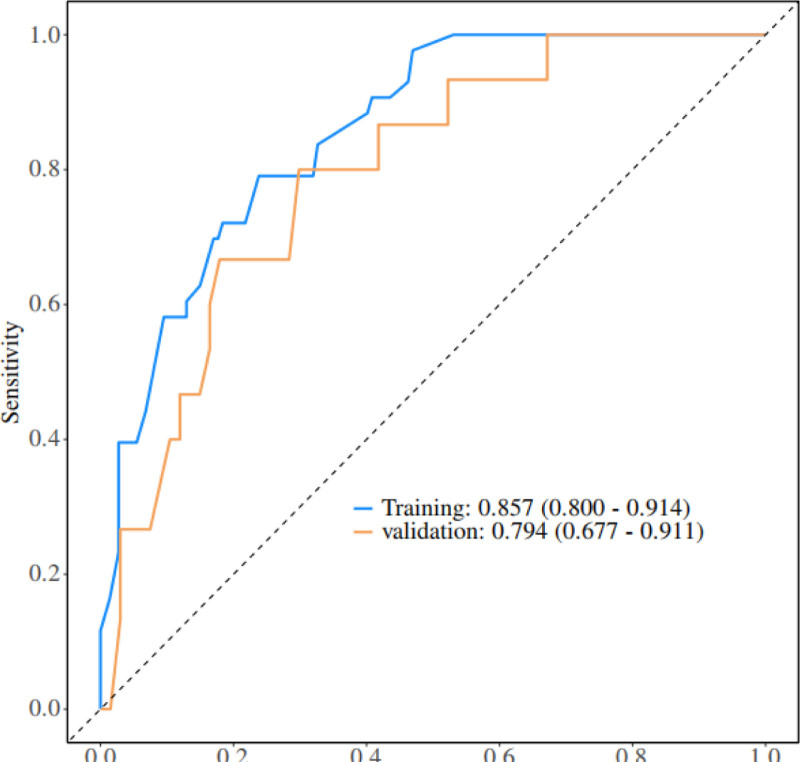
Validation of nomograms: ROC curves. ROC = receiver operating characteristic.

**Figure 4.**
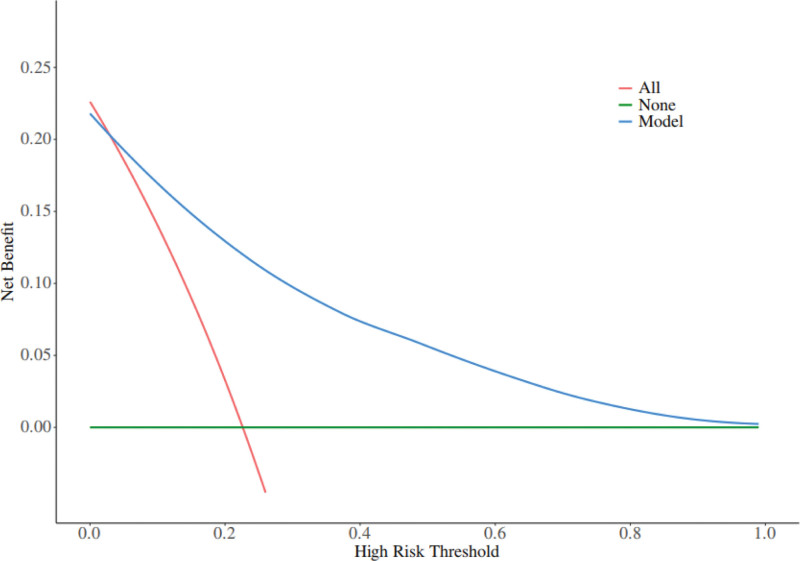
Decision curves in nomogram models.

Figure 2 for details. The nomogram has a receiver operating characteristic curve for the training set with an area under the curve (AUC) of 0.888 (95% confidence interval [CI] = 0.846–0.929) and a receiver operating characteristic curve for the validation set with an AUC of 0.853 (95% CI = 0.780–0.925), see Figure 3 for details. It shows that the nomogram has a good differentiation between those at high risk of developing PIAD after colostomy for colorectal cancer. The decision curve for this nomogram shows that the model provides more net benefits than the “all intervene” or ‘none intervene’ strategies when the threshold probability of an individual is >0.05 in this column. This finding suggests that this nomogram model has a good clinical application in predicting the development of PIAD after surgery in patients with colostomy for colorectal cancer, as detailed in Figure 4.

## 4. Discussion

Globally, the incidence rate of colorectal cancer continues to climb and remains high, and it has become one of the major malignant tumors that seriously threaten human health. As a key link in the comprehensive treatment system of colorectal cancer, stoma effectively relieves intestinal obstruction and improves intestinal function by establishing an artificial anus in the abdominal wall, which brings hope for many patients to extend their lives and improve the quality of life.^[13–15]^ Clinical data show that a significant proportion of stoma patients are afflicted by PIAD, which not only increases their pain, but also greatly affects their quality of life and recovery process. Despite previous studies, existing methods for predicting PIAD have significant shortcomings, making it difficult to accurately identify high-risk groups. In this context, it is urgent to carefully construct and validate a reliable PIAD prediction model, and this study will make every effort to fill this critical gap from theoretical analysis to clinical practice.

### 4.1. An in-depth review of PIAD risk factors for colorectal cancer

The data in this study showed that the incidence of PIAD was significantly lower in patients who attended the stoma clinic regularly than in those who did not. This is based on the fact that stoma clinic professionals are able to detect subtle changes in the stoma and surrounding skin in a timely manner with their experience and expertise. From the perspective of the impact mechanism, regular review can ensure that patients receive standardized stoma care instructions, such as the correct cleaning method and the timing of stoma appliance replacement. Healthcare professionals can also adjust the care plan according to the patient’s individual situation and correct the patient’s incorrect operation in the process of stoma care in a timely manner, which effectively reduces the risk of PIAD caused by improper care.^[16]^ In addition, this study clearly demonstrates that the stoma site is closely related to the occurrence of PIAD. The anatomical structure of the stoma site varies from one stoma site to another, which has a significant impact on the manner in which the excreta come into contact with the surrounding skin and the degree of irritation.^[17]^ For example, ileostomy discharge is mostly liquid and rich in digestive enzymes, and is more irritating to the skin around the stoma due to the relatively frequent discharge of fecal matter as a result of faster peristalsis at the end of the ileum. In contrast, colostomy drainage is thicker and less irritating.^[8,18,19]^ In addition, when the stoma is located in a skin fold, excretions tend to accumulate and do not favor a tight fit of the stoma appliance, further increasing the likelihood of PIAD.^[20]^ Secondly, poor sump fit was identified as an independent risk factor for PIAD. In this study, it was found that poorly fitted sumps were prone to leakage of excreta through follow-up observation of patients’ use of stoma appliances. From the perspective of mechanics, when the sump is not tightly fitted to the skin, excreta will leak out of the gap under pressure, continuously irritating the surrounding skin. At the same time, the non-fitting chassis will rub against the skin during the patient’s daily activities, destroying the skin barrier function and making the skin more susceptible to external irritants, thus inducing PIAD.In addition to that, hypoproteinaemia showed a significant correlation with the occurrence of PIAD in this study. In addition, hypoproteinaemia was significantly associated with the development of PIAD in this study. When the serum protein level is lowered, the nutritional status and immune function of the body are affected. Low protein leads to reduced repair and regeneration of skin tissue and weakened skin barrier function. Physiologically, the skin lacks sufficient protein to maintain normal cellular structure and function, making it more sensitive to irritants in the excreta and more prone to inflammatory reactions even under normal excreta, thus increasing the risk of PIAD.^[21]^ Finally, the patient’s anxiety state has been shown to be one of the independent risk factors for PIAD. In this study, the psychological state of patients was quantified and analyzed with the help of psychological assessment scales. The dysfunction of the patient’s autonomic nervous system in the state of anxiety can lead to abnormal vasoconstriction and diastolic function of the skin, affecting the blood circulation and metabolism of the skin. At the same time, anxiety may interfere with the patient’s acceptance and implementation of stoma care knowledge, reducing the patient’s ability to care for themselves, making the chances of inappropriate stoma care more likely, and ultimately increasing the likelihood of PIAD.^[22,23]^

The PIAD prediction model for colorectal cancer constructed in this study was analyzed by multifactorial logistic regression analysis, which identified regular outpatient review of the stoma, stoma site, sump fit, hypoproteinemia, and anxiety status as independent predictors, and the weights of each factor were specified. The model scores the clinical characteristics of the patients according to the weights of the factors, and the total score corresponds to the value of the Risk axis, i.e., the probability of a patient’s postoperative PIAD. As internally verified by the R software Bootstrap method with 1000 replicate samples, the calibration curve was close to the ideal curve with an AUC of 0.888 (95% CI = 0.846–0.929) for the training set and 0.853 (95% CI = 0.780–0.925) for the validation set. Decision curves showed good clinical application of the model, validating the validity and reliability of the model predictions.

### 4.2. Comparative analysis with the current state of domestic and international research

In the field of PIAD prediction in colorectal cancer, many studies have been carried out by previous researchers and achieved certain results. Overseas, Europe and the United States started earlier, and some studies focused on the association between the physicochemical properties of stoma excreta and the occurrence of PIAD. For example, through long-term tracking of patients with stoma, it was found that the high concentration of digestive enzymes in excreta was one of the key factors triggering PIAD, and then it was proposed that PIAD could be prevented by adjusting the dietary structure and reducing the irritation of excreta. However, such studies only focus on a single factor, ignoring other potential influences such as individual differences in patients, nursing behaviors and psychological status, making it difficult to comprehensively predict the risk of PIAD.^[24]^ In comparison, the PIAD prediction model constructed in this study demonstrated significant advantages. In terms of the comprehensiveness of risk factor identification, it breaks through the limitations of the previous study, which only focuses on a single or a few factors. In this study, several key factors such as regular stoma clinic review, stoma site, sump fit, hypoproteinaemia and anxiety status were considered. Among other things, regular stoma clinic reviews reflect the importance of continuity and regularity of nursing behavior for PIAD prevention; differences in stoma sites determine the natural characteristics of excreta skin irritation; and sump fit directly affects the degree of excreta skin contact and irritation; Hypoproteinaemia reflects the influence of the nutritional status of the patient on the skin barrier function; the anxiety state reveals the association between the patient’s mental factors and the occurrence of PIAD on a psychological level. The comprehensive inclusion of multiple factors makes the model more relevant to the complex and changing clinical reality. In terms of prediction accuracy, this study used multifactorial logistic regression analysis to determine the weights of each factor, and internal validation was carried out by repeating the sampling 1000 times through the Bootstrap method in R software. The results show that the calibration curve is close to the ideal curve with an area under the curve (AUC) of 0.888 (95% CI = 0.846–0.929) for the training set and 0.853 (95% CI = 0.780–0.925) for the validation set. This data suggests that the present model is able to identify high-risk patients with PIAD more accurately and reduce misclassification and underclassification compared to previous studies. These data show that, compared to previous research, the current model is more accurate in identifying high-risk PIAD patients, reducing both misdiagnosis and missed diagnoses.

There are many challenges in translating previous research findings into clinical practice, as the lack of comprehensive and accurate predictive models makes it difficult for healthcare professionals to take effective preventive measures at an early stage.^[25]^ The model constructed in this study provides clinicians with an intuitive and actionable risk assessment tool by quantitatively scoring various factors to derive the probability of postoperative PIAD in patients. Based on the results of the model, doctors can formulate personalized prevention and intervention plans for patients, such as strengthening the education for patients with poor compliance to regular review and adjusting the nutritional support plan for patients with hypoproteinemia, which greatly improves the efficiency and quality of clinical care, and has a high clinical value.

### 4.3. Model strengths, research limitations, and prospects

The PIAD prediction model for colorectal cancer constructed in this study shows significant positive significance in clinical practice. Firstly, it is able to accurately identify patients at high risk of PIAD at an early stage. Through the comprehensive analysis of several key factors, such as regular stoma clinic review, stoma site, chassis fit, hypoproteinemia, and anxiety status, the model can quickly screen out individuals with high risk of PIAD. For example, in a clinical practice case, a new patient undergoing stoma surgery was assessed by the model as having a stoma site in an irritable area with hypoproteinaemia and a high anxiety score, and the model predicted a significantly higher than average probability of PIAD. Based on this accurate prediction, healthcare professionals promptly formulated an intensive care plan for this patient, including increasing the frequency of stoma clinic review, adjusting the dietary structure to improve nutritional status, and providing professional psychological counseling, which effectively reduced the risk of PIAD. This model provides a clear direction of intervention for healthcare professionals. In the past, clinical care mostly relied on empirical judgement and lacked precise guidance, whereas the model clearly indicates the degree of influence of each risk factor, enabling healthcare professionals to target their work. For patients with increased risk due to poor sump fit, caregivers can focus on strengthening stoma appliance wearing guidance; For patients whose anxiety triggers an increased risk of PIAD, timely psychological intervention is provided. In this way, the nursing efficiency was greatly improved and the waste of resources caused by blind nursing was avoided. At the same time, through early and effective intervention, the incidence of PIAD was reduced, the pain caused by dermatitis was reduced, the prognosis of patients was improved, and the quality of life of patients was significantly enhanced.

Despite the results of this model in PIAD prediction, there are inevitably some limitations. In the prediction of special cases or rare situations, the model capability is somewhat insufficient. For example, for patients with rare genetic skin diseases who also underwent colostomy for colorectal cancer, the conventional risk factors included in the model could not fully reflect the risk of PIAD occurrence due to the specific underlying pathological state of the skin of these patients, resulting in potentially biased prediction results. Potential bias also exists in the data collection process. The data in this study were mainly obtained from some medical institutions, and the sample may have certain limitations in terms of geography, economic level of patients and accessibility of medical resources, so it cannot fully represent all groups of patients with colostomy for colorectal cancer. This may lead to an impact on the model’s prediction accuracy for patients in different regions and under different medical conditions when the model is promoted for application. In addition, patient care concepts, professionalism of healthcare professionals, and medical equipment vary greatly in different healthcare environments, and the applicability of the model in these complex and diverse healthcare environments varies, making it difficult to ensure optimal prediction efficacy in all scenarios. Since the pathogenesis of PIAD is still unknown, in-depth basic research is needed in the future. On the one hand, efforts should be made to investigate the interaction between risk factors. For example, it is important to investigate how hypoproteinemia and anxiety interact to synergistically increase the risk of PIAD; To dissect the circumstances under which stoma site factors and chassis fit can have a superimposed effect and aggravate skin irritation. On the other hand, to study in depth the influence of these factors on the physiopathological processes of the skin through molecular biological pathways.

## 5. Conclusions

In this study, after rigorous analysis and research, we clarified regular stoma clinic review, stoma site, sump fit, hypoproteinemia and anxiety state, as independent risk factors for PIAD in colorectal cancer, and constructed a prediction model. The model integrates clinical observations, refines the knowledge of the pathogenesis of PIAD, and provides new ideas for in-depth investigation of its pathophysiology. In practice, it helps clinicians to identify patients at high risk of PIAD at an early stage, so that targeted prevention and intervention strategies can be formulated. Specifically, it includes reminding patients to have regular checkups, selecting appliances according to the fit of the stoma site and the chassis, adjusting the nutritional regimen for patients with hypoproteinemia, and providing psychological counseling for patients with anxiety, so as to reduce the risk of PIAD. This effectively improves the quality of stoma care, reduces the incidence of PIAD, and improves the quality of life and prognosis of patients.

## Author contributions

**Project administration:** Fei An.

**Writing – original draft:** Fei An, Lin Gui.

**Writing – review & editing:** Fei An, Yuting Li, Minjing Cheng.
